# Estimating Antigen Test Sensitivity via Target Distribution Balancing: Development and Validation Study

**DOI:** 10.2196/68476

**Published:** 2025-10-20

**Authors:** Miguel Bosch, Adriana Moreno, Raul Colmenares, Jose Arocha, Sina Hoche, Auris Garcia, Daniela Hall, Dawlyn Garcia, Lindsey Rudtner, Nol Salcedo, Irene Bosch

**Affiliations:** 1 IDX20 Inc, 166 Clinton Road, Brookline, MA, United States, 1 6177347745; 2 Info Analytics Innovations LLC, Houston, TX, United States

**Keywords:** antigen test, COVID-19, positive percent agreement, real-world data, sensitivity estimation, PPA

## Abstract

**Background:**

Sensitivity—expressed as percent positive agreement (PPA) with a reference assay—is a primary metric for evaluating lateral-flow antigen tests (ATs), typically benchmarked against a quantitative reverse transcription polymerase chain reaction (qRT-PCR). In SARS-CoV-2 diagnostics, ATs detect nucleocapsid protein, whereas qRT-PCR detects viral RNA copy numbers. Since observed PPA depends on the underlying viral load distribution (proxied by the number of cycle thresholds [Cts], which is inversely related to load), study-specific sampling can bias sensitivity estimates. Cohort differences—such as enrichment for high- or low-Ct specimens—therefore complicate cross-test comparisons, and real-world datasets often deviate from regulatory guidance to sample across the full concentration range. Although logistic models relating test positivity to Ct are well described, they are seldom used to reweight results to a standardized reference viral load distribution. As a result, reported sensitivities remain difficult to compare across studies, limiting both accuracy and generalizability.

**Objective:**

The aim of this study was to develop and validate a statistical methodology that estimates the sensitivity of ATs by recalibrating clinical performance data—originally obtained from uncontrolled viral load distributions—against a standardized reference distribution of target concentrations, thereby enabling more accurate and comparable assessments of diagnostic test performance.

**Methods:**

AT sensitivity is estimated by modeling the PPA as a function of qRT-PCR Ct values (PPA function) using logistic regression on paired test results. Raw sensitivity is the proportion of AT positives among PCR-positive samples. Adjusted sensitivity is calculated by applying the PPA function to a reference Ct distribution, correcting for viral load variability. This enables standardized comparisons across tests. The method was validated using clinical data from a community study in Chelsea, Massachusetts, demonstrating its effectiveness in reducing sampling bias.

**Results:**

Over a 2-year period, paired ATs and qRT-PCR–positive samples were collected from 4 suppliers: A (n=211), B (n=156), C (n=85), and D (n=43). Ct value distributions varied substantially, with suppliers A and D showing lower Ct (high viral load) values in the samples, and supplier C skewed toward higher Ct values (low viral load). These differences led to inconsistent raw sensitivity estimates. To correct for this, we used logistic regression to model the PPA as a function of Cts and applied these models to a standardized reference Ct distribution. This adjustment reduced bias and enabled more accurate comparisons of test performance across suppliers.

**Conclusions:**

We present a distribution-aware framework that models PPA as a logistic function of Ct and reweights results to a standardized reference Ct distribution to produce bias-corrected sensitivity estimates. This yields fairer, more consistent comparisons across AT suppliers and studies, strengthens quality control, and supports regulatory review. Collectively, our results provide a robust basis for recalibrating reported sensitivities and underscore the importance of distribution-aware evaluation in diagnostic test assessment.

## Introduction

Antigen tests (ATs) have been a common tool utilized to provide evidence for diagnosis and health care decisions and have been used as such for several decades [1-4]. During the COVID-19 pandemic, the worldwide use of ATs demonstrated relevance for disease monitoring and diagnosis of new cases [5,6]. ATs are rapid, economical, and portable; can be self-administered; are quick to develop; and provide direct evidence for the presence of the pathogen in the tested sample—this combination is not equaled by more sophisticated laboratory tests. Due to its increasing use, it is important to accurately evaluate the AT performance for quality and regulatory control.

The common statistic used to evaluate the positive agreement performance of ATs has been sensitivity, calculated over a set of samples known to be positive by a gold standard reference. However, the sensitivity of ATs is known to be strongly dependent on the sample viral load [7-9]. Hence, the sensitivity per se is not an appropriate measure of the AT positive agreement performance because it is largely dependent on the distribution of the viral load of statistical support (ie, the set of samples used to calculate the statistic). Instead, a description of the percent positive agreement (PPA) as a function of viral load is a more accurate measure of positive agreement performance. The PPA function (PPAf) is commonly calculated with a logistic regression of the binary test result on positive agreement (1=agreement, 0=disagreement) against a variable related to the viral load. In the case of COVID-19, the viral load is commonly measured with the quantitative reverse transcription polymerase chain reaction (qRT-PCR) cycle threshold (Ct) result [10,11]; hence, the PPA is a function of the Cts.

Once the PPAf of a given AT supplier’s product has been estimated from collected data in real-world application conditions, it is straightforward to estimate the expected sensitivity for any given Ct distribution or Ct sample set. This is particularly useful for equalizing the expected sensitivity to a common standard or reference distribution of Cts for a comparison of the performance across suppliers for product quality or regulatory purposes. This process removes the bias introduced into sensitivity by the circumstantial uneven representation of viral load in the statistical support (ie, the data used to calculate the statistic).

Common methods used to calculate the sensitivity of an AT product, whether for regulatory compliance or clinical diagnostic research purposes, typically involve collecting real-world test results paired with the qRT-PCR gold standard. However, in the case of lateral-flow ATs, sensitivity uncertainty does not adhere to a straightforward Bernoulli process, as the underlying positive agreement probability is not constant and is instead conditioned by viral load. To accurately calculate sensitivity, it would be necessary to segment the collected samples based on the most influential variable affecting underlying probability, using a standard reference histogram. In our case, the influential variable is the viral load, measured as Cts. Yet, implementing such a process in the field with real-world data would be cumbersome and would require a much larger dataset. The proposed balancing method overcomes this challenge by adjusting the raw sensitivity calculation to any desired standardized reference distribution of the viral load, without the need for extended data collection and segmentation.

## Methods

### Study Description

We conducted a study of AT use in real-world conditions in the city of Chelsea, Massachusetts, during the years 2022‐2023. The objectives of the study were multifold: (1) performing frequent COVID-19 testing at 2 vulnerable population sites (elderly housing), (2) evaluating the performance of ATs from different suppliers in the laboratory and in the real-world context, (3) collecting longitudinal (time series) AT data for qRT-PCR positive samples, and (4) implementing a digital AT data collection platform.

The participants of the study were enrolled after consent. The participant was provided with a single self-testing AT of any of the available 4 participating suppliers. The tests were home tests, and data were self-logged by the participants into an internet-based informatics platform. The participants registered the AT results (their own assessment) and uploaded a photograph of the test after completion (15 min). The fraction of positive AT tests was followed daily with paired qRT-PCR testing. A random number of negative ATs were also analyzed by PCR.

Support personnel were available on scheduled days throughout the week within the community to provide devices, training, and participant follow-up. This minimized potential confounding factors related to test interpretation and self-reporting via the app. In addition to textual reports, participants uploaded an image of their test result, allowing our team to perform retrospective verification. Discrepancies between self-reported results and staff-reviewed interpretations were found to be negligible, occurring in 5 eye assessments of 500 positive tests.

The data analyzed come from ATs provided by 4 different suppliers, labeled A through D, and the corresponding qRT-PCR test results, all of which were processed at a Clinical Laboratory Improvement Amendments–certified laboratory using the same RNA extraction as well as PCR protocol. The total negative qRT-PCR tests were 57 for A, 91 for B, 145 for C, and 114 for D, and positive qRT-PCR tests were 211 for A, 156 for B, 84 for C, and 43 for D. Each participant was tested with a single brand (ie, AT supplier), so the distribution of viral load could be different between the data collected for each brand. These data were used to calculate AT performance statistics and demonstrate the methodology for distribution-balanced sensitivity according to a reference standard distribution. Lineage annotations used in this study for laboratory assays were Delta comparator (B.1.617.2/AY sublineages) using the US WA 1/1 isolate and Omicron (BA.5). Among Chelsea study participants in 2022-2023, we detected BA.1.1, BA.2, BA.2.12.1, BA.5, BA.5.1, BA.5.2, BA.5.2.1, BQ.1.1, BQ.1.1.4, BQ.1.1.5, BQ.1.14, and XBB.1.5. Clinical sample sequences were generated at the Broad Institute.

### Ethical Considerations

This study was reviewed and approved by the Advarra Institutional Review Board (IRB) for protocol “Center of Complex Interventions – IDx20-001, Community frequent antigen testing to monitor COVID-19 in senior public housing setup” (Pro00059157). The most recent continuing review approval was granted on November 13, 2023, with approval through November 13, 2024. Advarra is registered with the Office for Human Research Protections/Food and Drug Administration (IRB #00000971) and conducts reviews in accordance with US HHS 45 CFR 46 and FDA 21 CFR 50/56. All procedures adhered to institutional and national ethical standards and the World Medical Association Declaration of Helsinki. In accordance with JMIR Publications requirements, the IRB review outcome is explicitly reported here.

Before any study procedures, participants were informed of the study purpose, procedures, potential risks and benefits, data uses, and their right to withdraw without penalty. Written informed consent was obtained from each participant using IRB-approved consent materials. No monetary compensation was given to participants. All study personnel completed human-subjects protection training prior to participant interaction.

Data were collected and stored under IRB-approved procedures to protect privacy and confidentiality. Only deidentified or aggregated data are presented in this study; no identifiable personal information is reported.

Any protocol amendments, consent-form changes, or reportable events (eg, unanticipated problems; adverse device effects; or protocol deviations that could affect participant rights, safety, or data integrity) were submitted to Advarra in accordance with IRB requirements before implementation.

### PPA Function

As commonly used, AT operative reading involves steps of device reaction to the nasal swab sample, a waiting time after the sample is deposited, an observation using the naked eye, and interpretation of the results by the user. For all kits utilized (eg, cassettes), the user observed the presence or absence of a colored line in a test area (*test band*) on a nitrocellulose strip of the lateral-flow test. The result is considered *positive* when the test band visualized (a color band) and can be distinguished from the no color or white background even if the color signal is faint, while a *negative* result is when the test band cannot be visualized by the user. For performance statistics, the result of the AT provided to the user is compared to the gold standard reference test [12], a COVID-19 qRT-PCR test conducted in a state-approved clinical laboratory.

For the positive agreement analysis of each AT supplier dataset, we compare only the AT results that have a paired positive qRT-PCR result (ie, having a positive qRT-PCR result in a parallel swab sample taken the same day and time as the AT swab sample). For the logistic regression analysis, we identified the AT results with a binary variable: 1 for a positive result (agreement with the standard test) and 0 for a negative result (disagreement with the standard test). The outcome of the user assessment can be described by a binary random variable. We modeled the PPAf with a logistic function, having the qRT-PCR Ct as the function domain (ie, the independent variable). Logistic regression is a well-known analysis to estimate the probability as a function of a dependent variable [13]. It has been used to describe the probability of positive agreement in ATs [14].

In addition to estimating the PPAf that characterized each AT supplier, the regression also accounts for the uncertainties of the probability function and parameters. We implement the logistic regression with a Bayesian approach, combining the objectives of (1) fitting the binary observed data and (2) honoring the Clopper-Pearson binomial confidence limits at the raw Ct data sensitivity prediction. Hence, the posterior model uncertainty description ensures compliance with the Clopper-Pearson confidence limits for the sensitivity at the raw Ct data. The numerical calculations are performed by Markov chain Monte Carlo methods.

The logistic model is a well-established method for modeling binary outcomes in which the probability, $p\left(x\right)$, varies with a predictor variable, *x*, particularly when the probability increases monotonically with *x*. It assumes that the log-odds of the outcome (ie, the logit transformation of the probability) is a linear function of the predictor. Compared to the traditional approach to raw sensitivity estimation—which treats test outcomes as the result of a Bernoulli process with constant (homogeneous) probability—the logistic model offers a first-order improvement by accounting for the dependence of the outcome probability on the target concentration. Although alternative parametric models could be used to describe the relationship between probability and the predictor (eg, probit, splines), the logistic model provides a widely accepted and practical framework for improving sensitivity estimation accuracy. Logistic and probit models both yield nearly identical monotone dose-response curves; we selected the logistic model for its interpretability in terms of log-odds, its ability to directly estimate the concentration at which PPA=0.5, and its widespread use in diagnostics. Spline approaches are not appropriate in this context, as they do not satisfy the boundary conditions of approaching 0 at the lower end and 1 at the upper end of the concentration axis.

### Distribution-Balanced Sensitivity Method

The sensitivity, *s*, is the fraction of the positive agreement cases divided by the total positive cases in the experimental gold standard results (eg, collected real-world AT binary data on positive qRT-PCR cases). Based on the PPAf characterized for each AT supplier data, we can estimate the sensitivity for any set of Ct cases. Let us consider that the experimental data for a given AT supplier involves N cases with qRT-PCR cycle counts, $x=\left\{x_{1},x_{2},\ldots x_{n},\ldots ,x_{N}\right\}$. The expected value of the sensitivity is the average of the PPAf, $p\left(x\right)$, over the cases:


(1) $$E(s)=\frac{1}{N}\sum_{1}^{N}p\left(x_{n}\right)$$

Likewise, the expected sensitivity over a data support with any Ct probability density function (PDF), g(x), is given by the probability product integration:

. (2) $$E(s)=\frac{1}{xf-x0}\int_{x0}^{xf}p\left(x\right)g\left(x\right)dx$$

Equations 1 and 2 use the PPAf to calculate the expected sensitivity over a specific Ct data or distribution. Adequate comparison of sensitivity across different AT datasets requires a transformation of raw sensitivity (ie, calculated from the observed data) to the expected sensitivity over a common reference of Ct data values, or a Ct support distribution (ie, histogram or PDF). Considering observed binary data for several AT suppliers, our proposed process to equalize the sensitivity support involves (1) estimating the PPAfs by logistic regression of the observed AT binary data for each one of the supplier’s datasets; (2) defining a common reference Ct distribution by a PDF, $g\left(x\right)$; and (3) calculating for each AT supplier dataset the estimated sensitivity over the common reference Ct support by equation (2).

Equation 2 can be evaluated by discretizing the Ct domain. Alternatively, it can be computed by Monte Carlo integration, drawing Ct realizations from the target distribution and applying equation 1 to each draw. To balance viral load distributions across assays, we select a reference Ct sample set as the empirical Ct distribution from supplier A and evaluate all other suppliers over this common range. The procedure is as follows: (1) for each nonreference supplier, fit a logistic regression for PPA as a function of Ct using the observed binary AT outcomes and (2) compute expected sensitivity by averaging the fitted PPA over supplier A’s Ct values via equation 1.

The described viral load balance processes removed the effect of the Ct distribution on sensitivity, providing a common base for comparison and evaluation of the test performance.

Table 1 presents a glossary of terms and their corresponding meanings.

**Table 1.** Glossary of relevant terms and corresponding meanings.

Term	Definition
Antigen test (AT)	Lateral flow antigen test
Target	Specific analyte that the test is designed to detect (protein present in a biological matrix sample)
Cycle threshold (Ct)	qRT-PCR^a^ cycle at which the fluorescence signal crosses a set threshold above background
Target concentration	It is the concentration of the target protein, expressed by cycle thresholds or ng mL^–1^, or by plaque forming units of virus mL^–1^
Target concentration distribution	The distribution of target concentrations among the tested population; when grouped into concentration ranges, it can be represented as a histogram.
Sensitivity	Proportion of positive cases detected by the antigen tests according to the reference qRT-PCR gold standard. It represents the percent positive agreement.
Percent positive agreement (PPA)	In a Bernoulli process, it is the probability that the test outcome is positive; in the context of antigen tests, this probability varies with the target concentration.
Percent positive agreement function (PPAf)	It is a function assigning the value of the probability of positive agreement for a given target concentration.
Distribution-balanced sensitivity	The modeled value of the sensitivity at a selected (balanced) cycle distribution distribution different from that of the real-world dataset.
Reference distribution	A distribution of the target concentration that is adopted as reference to model the sensitivity.

a qRT-PCR: quantitative reverse transcription polymerase chain reaction.

## Results

### Raw Positive Agreement Statistics

This section describes the basic performance statistics of the ATs of the 4 suppliers analyzed, and the estimated PPAfs, based on the binary data collected from the Chelsea study [15]. The agreement matrix was determined for each supplier AT, and the common performance agreement fractions were calculated: sensitivity, specificity, positive prediction, negative prediction, and total prediction. Table 2 displays the basic performance statistics for each one of the test suppliers, including the Clopper-Pearson 95% confidence limits [16].

**Table 2.** Basic performance statistics for COVID-19 in vitro diagnostics suppliers A, B, C, and D.

Supplier		Positive agreement cases, n	Total cases, n	Value	Lower 95% confidence limit	Upper 95% confidence limit
A						
Sensitivity		177	211	0.84	0.78	0.89
Specificity		55	57	0.96	0.88	1.00
Positive prediction		177	179	0.99	0.96	1.00
Negative prediction		55	89	0.62	0.51	0.71
Total agreement		232	268	0.87	0.82	0.90
B						
Sensitivity		117	156	0.75	0.67	0.82
Specificity		90	91	0.99	0.94	1.00
Positive prediction		177	118	0.99	0.95	1.00
Negative prediction		90	129	0.70	0.61	0.77
Total agreement		207	247	0.84	0.79	0.88
C						
Sensitivity		55	85	0.65	0.54	0.75
Specificity		144	144	1.00	0.97	1.00
Positive prediction		55	55	1.00	0.94	1.00
Negative prediction		144	174	0.83	0.76	0.88
Total agreement		199	229	0.87	0.82	0.91
D						
Sensitivity		35	43	0.81	0.67	0.92
Specificity		107	114	0.94	0.88	0.97
Positive prediction		35	42	0.83	0.69	0.93
Negative prediction		107	115	0.93	0.85	0.97
Total agreement		142	157	0.90	0.88	0.95

**Figure 1.**
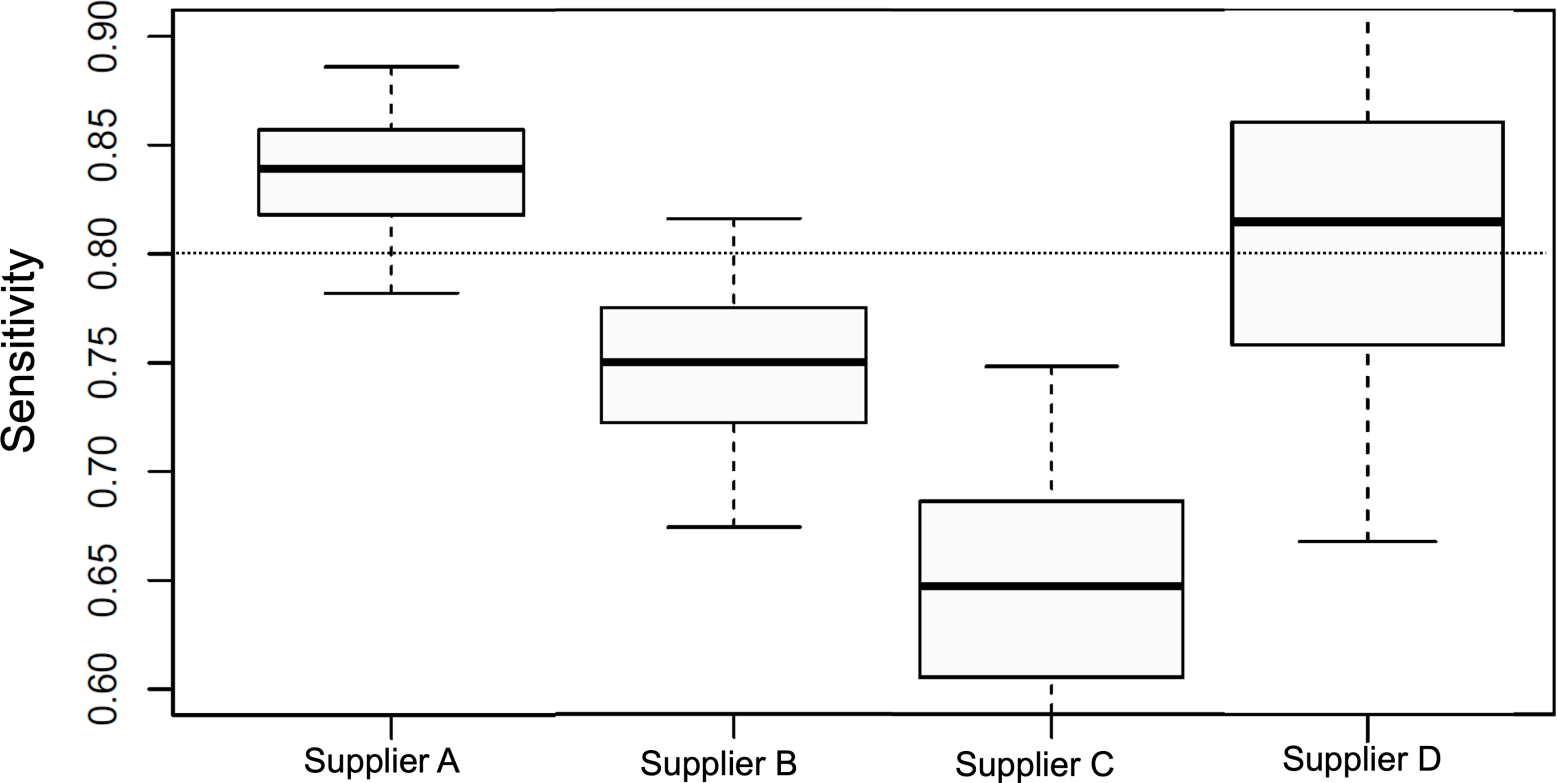
Raw sensitivities resulting from the real-world Chelsea study for lateral flow in vitro diagnostics suppliers A, B, C, and D. The boxes and whiskers indicate the median and 50% and 95% confidence limits calculated using the Clopper-Pearson statistical method. The horizontal dotted line across all histograms indicates the 0.8 sensitivity value, which is acceptable for in vitro diagnostics clinical performance according to regulatory standards at 0.8 sensitivity for COVID-19 antigen tests.

**Figure 2.**
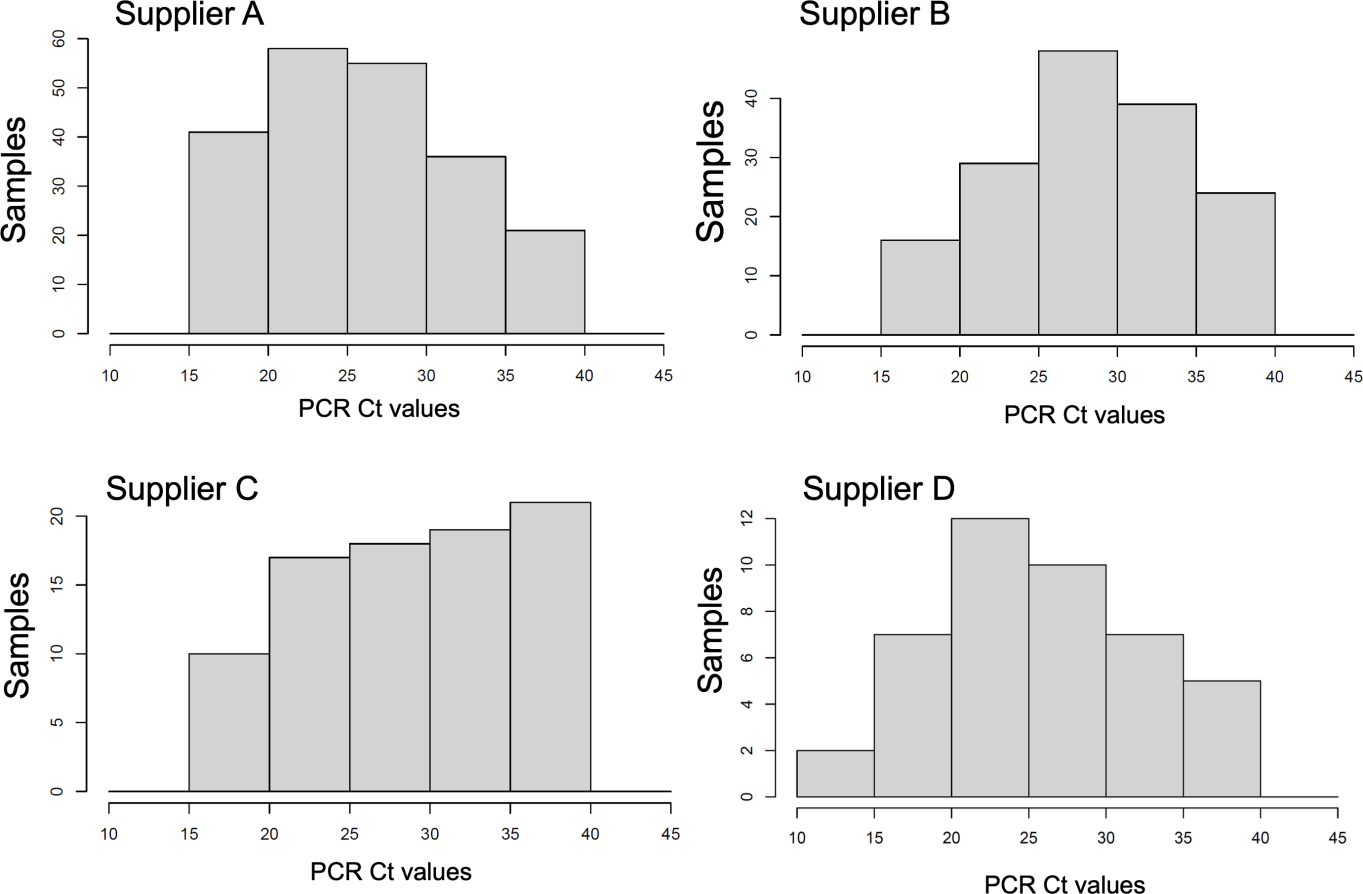
Histogram distributions of cycle thresholds (Cts) for antigen test suppliers A-D using quantitative reverse transcription polymerase chain reaction (qRT-PCR) Cts for each antigen test supplier’s dataset. Cts are the average of N and ORFab gene segments of SARS-CoV-2 and reported from Clinical Laboratory Improvement Amendments–certified laboratory using a PerkinElmer SARS-CoV-2 Food and Drug Administration–approved kit.

Sensitivities show large differences across the suppliers A, B, C, and D, with values 0.84, 0.75, 0.65, and 0.81, respectively. A comparison plot of the raw AT sensitivities for each supplier and confidence limits is shown (Figure 1). Differences are significant with a large departure of 19% (percentage points of the sensitivity) between suppliers A and C. However, the histograms of qRT-PCR Cts supporting the sensitivity calculations have marked differences across the suppliers (Figure 2). Note that suppliers A and D have a larger proportion of low Cts (large viral sample concentration). On the other hand, supplier C has a larger representation of large Cts (low viral sample concentration).

### Probability of Positive Agreement Functions

**Figure 3.**
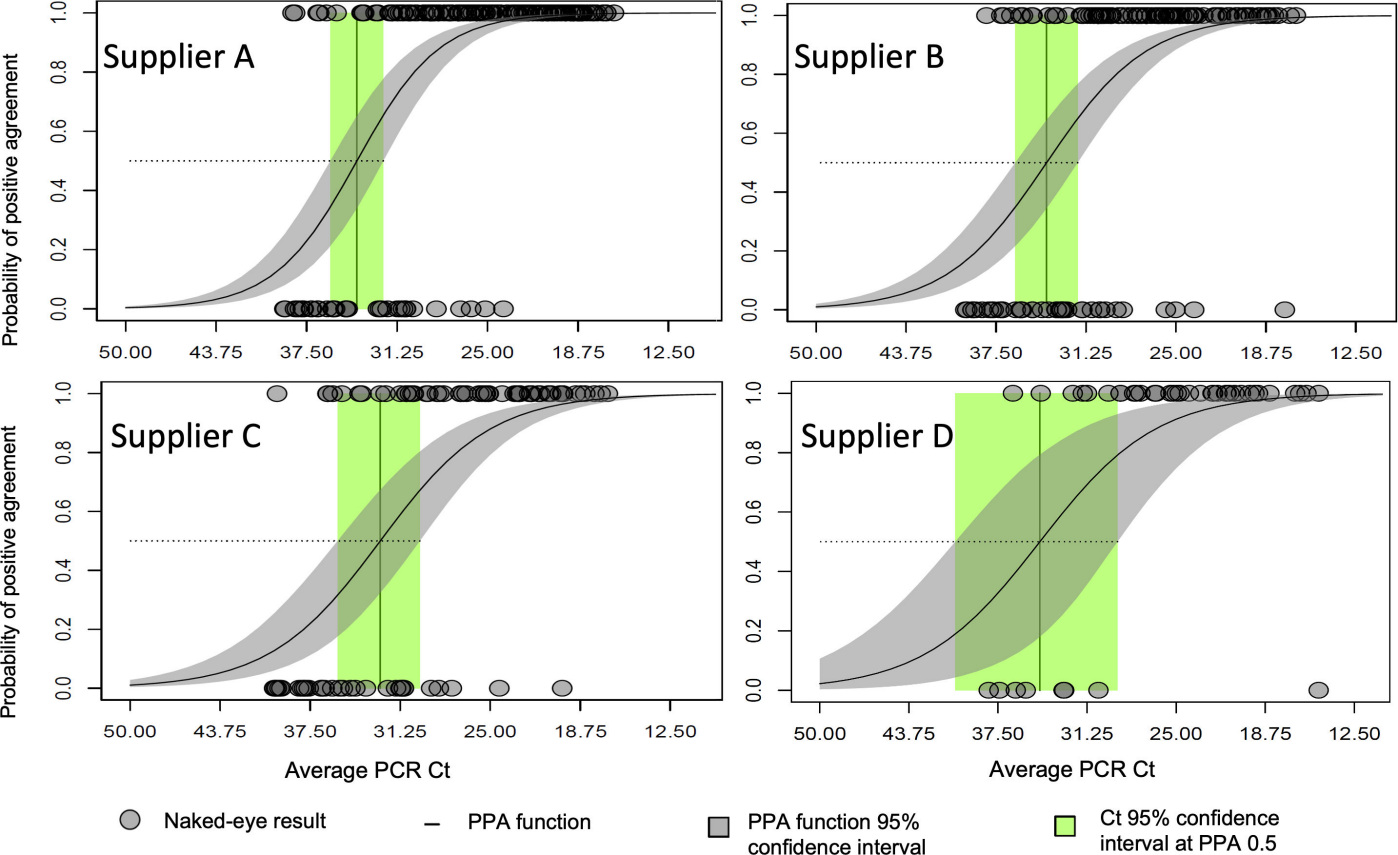
Positive percent agreement (PPA) as a function of the quantitative reverse transcription polymerase chain reaction (qRT-PCR) cycle thresholds (Cts) for naked-eye assessments of the Chelsea project participants after self-application of the antigen tests. Naked-eye assessments of the antigen test result are plotted in the vertical axis with value 1 for positive and 0 for negative. The PPA function is obtained by logistic regression of the binary naked-eye results and shows the strong dependency of the agreement probability with the qRT-PCR Cts.

Raw sensitivities (Figure 1) superpose the effects of the viral concentration support to the true performance of the ATs. Following our method, a first step to decouple the 2 effects is estimating the PPAf from the raw data of each AT supplier by logistic regression, as explained in the previous section. Figure 3 shows the binary data collected for each AT supplier plotted against the Cts and the estimated PPAf for each test supplier. The estimation of the PPAf by fitting the observed binary AT data also provides the description of the uncertainties in the PPAf. With the 95% confidence intervals of the PPAf, our formulation estimates the full distribution of the PPA conditioned to the Ct value.

The PPAfs for suppliers A and B are similar (Figure 3). Note that the Ct of median probability (ie, limit of detection at probability *P*=.50) and the slope of the line function are similar to one another. The PPAf for supplier C shows a slightly larger Ct at median probability and lower slope of the function. The PPAf for supplier D is also close to the A and B functions but shows a wider range of uncertainties, also expected from the smaller dataset supporting supplier D.

Several factors influence the estimation of the PPAf: the representation of the Ct range, the balance between positive and negative samples, and the overall sample size. Smaller sample sizes increase the uncertainty of the PPAf, as illustrated in the case of supplier D. However, in this case, the estimation remained reliable because the Ct range and the positive-negative representation were sufficiently well balanced. The Ct values in supplier D’s dataset are comparable to those observed in other supplier datasets. Similarly, the distribution of positive and negative samples is appropriate, with a higher frequency of negatives at lower viral loads (ie, higher Ct values), as expected. In contrast, datasets with limited coverage of the Ct range, underrepresentation of positives or negatives, or a distribution that fails to reflect the expected polarization, with negatives concentrated at higher Ct values and positives at lower Ct values, would not support a reliable estimation.

### Reference Distributions of Viral Load

**Figure 4.**
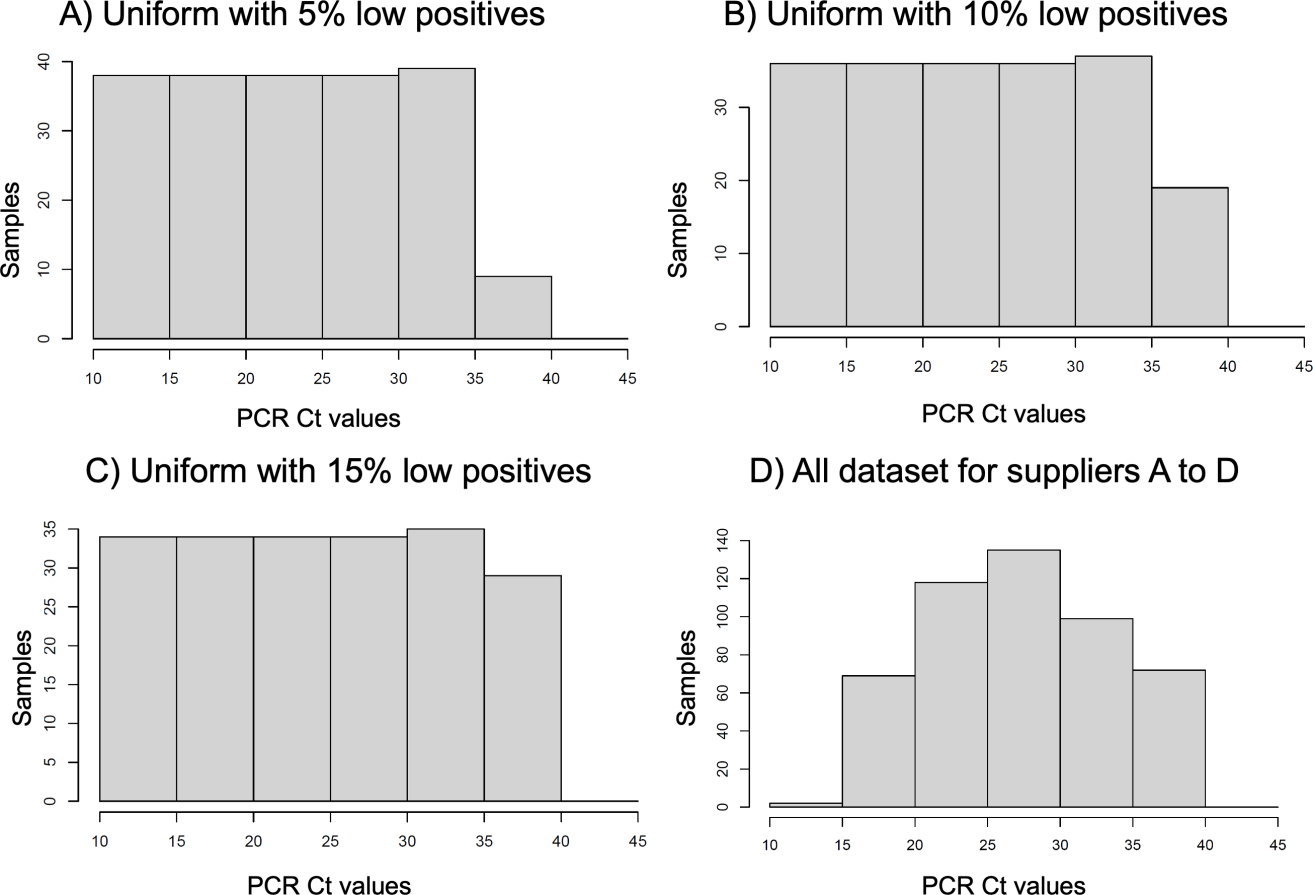
Comparison of selected reference distributions of the raw data. Histograms A, B, and C correspond to 200 dataset points with a range of low virus load (from 5% to 15% of sample at 35‐40 quantitative reverse transcription polymerase chain reaction [qRT-PCR] cycle threshold [Ct] range) and uniform distribution in the range of high-to-moderate virus load (10‐35 qRT-PCR Ct range). Histogram D corresponds to the overlap of the 4 antigen test in vitro diagnostics suppliers’ real-world data.

Due to the specific Ct value distributions, the comparison of raw sensitivities is biased by the uneven distribution of the Ct support. This is shown by the corresponding histograms (Figure 2). Utilizing the balance methodology, we computed the sensitivities of the ATs from the 4 suppliers across 4 distinct reference distributions of the Cts. The sample statistics of the PPA exhibit particular sensitivity to low positive samples, that is, those with low viral concentration. Consequently, we opt for a uniform distribution of qRT-PCR cycles spanning 10‐35 Cts, with variable proportions within the 35‐40 range, to underscore the significance of representing low-positive cases in the overall sample PPA (Figure 4A-C). Additionally, we employed the combined Ct distribution of all 4 tests as a reference, that is, the joint positive qRT-PCR Ct counts of the 4 suppliers’ data (Figure 4D).

We present 4 different Ct distributions to illustrate how Ct values, and therefore virus load, influence the adjusted sensitivity. The purpose of the distribution-balancing process is 2-fold: (1) to enable accurate comparison of AT sensitivities across studies and (2) to improve real-world sensitivity estimation. First, comparing sensitivities across studies becomes more reliable when distribution-balanced is referenced to a common distribution. Second, clinical studies often have limited numbers of cases and may not adequately reflect the real-world distribution of target concentrations observed in broader populations (eg, regional or national data). In this context, distribution-balanced sensitivity provides a closer approximation of real-world performance. For our clinical dataset, the distribution that best represents the broader population is the overall Ct dataset (Figure 4D), as it includes all cases pooled from the 4 devices.

### Sensitivities for the Reference Distributions

**Figure 5.**
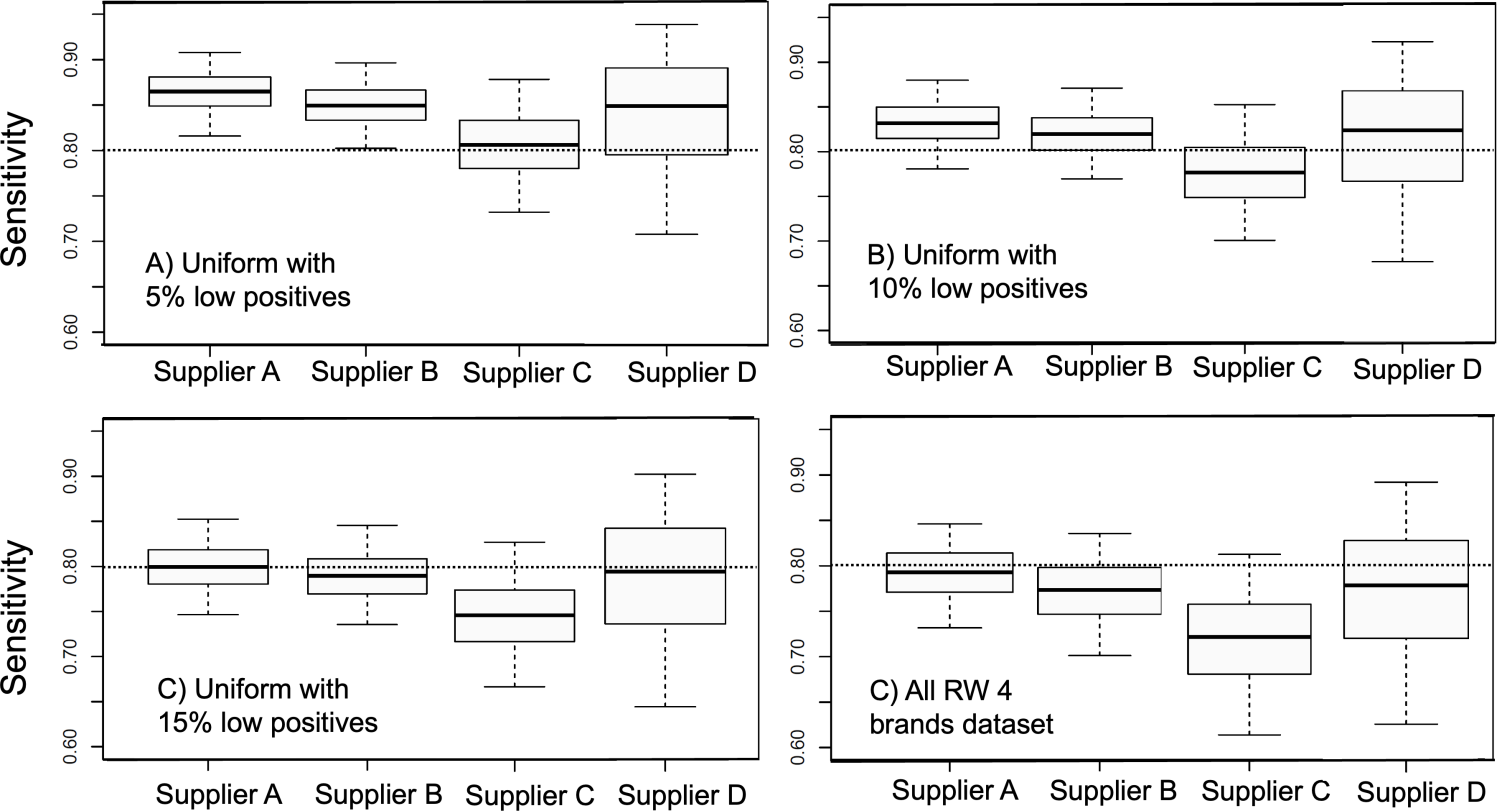
Sensitivities of antigen test brands calculated from reference distributions of cycle thresholds as shown in Figure 4. The boxes and whiskers indicate median and 50% and 95% confidence limits. Horizontal dotted line across each panel shows the threshold of 0.8 sensitivity acceptable for in vitro diagnostics clinical performance according to regulatory standards for COVID-19 antigen tests. RW: real world.

According to the described viral load balance method, we estimated the sensitivities of the ATs of the 4 suppliers (Figure 5) over each one of the reference distributions (Figure 4) utilizing the PPAf. It is interesting to compare the results shown (Figure 4) to the raw sensitivities previously calculated (Figure 1). Although the order of performance of the 4 suppliers has been preserved, in order of best to worst performances, the order was supplier A, B, D, and C. The sensitivity differences are smaller among suppliers once the effect of the source distribution is removed. The difference between suppliers A and C is only at 5% instead of the 19% for the raw sensitivity calculation. A large proportion of the raw sensitivity difference between these 2 suppliers originated from the overrepresentation of high viral concentration samples for supplier A and the overrepresentation of low viral concentration samples for supplier C.

Figure 5 shows the impact of the reference distributions of Ct values on the resulting sensitivity. The absolute value of the sensitivity shows a variation of over 8% difference across the different distributions—larger than the difference across the supplier sensitivities. In particular, the fraction of low virus concentration positives plays an important role, as expected: the PPAfs (Figure 3) show that the probability of positive agreement is very low for low positives (35‐40 Cts) in all suppliers. The indicated line at 0.8 sensitivity in Figure 5 helps to illustrate this point. With the reference distribution including 5% of low positives, the 4 suppliers have sensitivities over the 0.8 threshold. With the reference distribution including 15% of low positives, suppliers B and C are below the threshold, whereas suppliers A and D are borderline at the 0.8 threshold value. With the distribution that combines the 4 suppliers’ observed samples, all the suppliers are below the 0.8 threshold.

## Discussion

Accurate estimation of AT performance in real-world studies is often confounded by heterogeneous viral load distributions, sample collection conditions, and demographic factors [17]. In particular, the Ct values derived from qRT-PCR, which serve as a surrogate for viral concentration, exhibit considerable variability across study populations. This variability can bias sensitivity—or PPA—estimates when derived directly from unbalanced datasets.

To address the limitation, we introduce a mathematically grounded approach that estimates a PPAf via logistic regression and then recalculates sensitivity over a standardized reference Ct distribution. This method transforms raw sensitivity estimates into a harmonized metric that is independent of the original data’s viral load distribution, enhancing comparability across studies and diagnostic platforms. By modeling the entire range of Ct values, rather than focusing solely on predefined low-positive bins, our approach enables more comprehensive and statistically balanced evaluations. A reference distribution of qRT-PCR Ct values serves as a standardized representation of viral load across a target population, enabling consistent evaluation of diagnostic test sensitivity. Unlike raw distributions derived from individual clinical studies—which are subject to variability in recruitment timing, population demographics, testing strategies, and local epidemiology—a reference distribution is designed to reflect a controlled or representative viral load profile against which diagnostic performance can be compared. The reference Ct distribution may be empirically derived from large, well-characterized datasets collected during peak transmission periods or constructed synthetically based on known viral kinetics in the population. For example, an ideal reference might be a unimodal distribution centered around the Ct range associated with peak transmissibility and highest clinical relevance (eg, Ct 20‐30), or it might reflect the full spectrum of observed viral loads (eg, Ct 10‐40), weighted to mirror realistic clinical case presentations across settings. The purpose of applying such a reference is to enable adjusted sensitivity calculations that are independent of the viral load biases inherent in the original data. This is particularly important when comparing AT performance across different suppliers or studies, where raw sensitivities may differ simply due to the proportion of high- or low-Ct samples in each dataset. In the diagnostic settings, a broader distribution capturing both early and late stages of infection may be more appropriate. Ultimately, the choice of reference distribution must be consistent, allowing for harmonized sensitivity comparisons that reflect diagnostic utility across diverse real-world scenarios.

While regulatory frameworks have moved toward including “lower viral load specimens” in performance assessments to mitigate overestimation, these approaches often lack generalizability and do not account for the full spectrum of observed Ct values. Our methodology advances this paradigm by providing a distribution-based recalibration mechanism, capturing the full continuum of viral concentrations, and generating sensitivity estimates that are both internally consistent and externally comparable. Critically, FDA Emergency Use Authorization evaluation criteria anchored to qRT-PCR (including use of highly sensitive PCR comparators and mandated proportions of “low-positive” samples) shaped apparent clinical sensitivity and reinforced PCR-first policies for confirmation, which—despite PCR’s analytical advantages—dampened uptake of antigen testing for rapid, frequent screening where time-to-result drives transmission control.

Furthermore, genetic strain diversity, specific amino acid mutations in the SARS-CoV-2 nucleoprotein, and host disease–related comorbidities and immunological factors [18,19] may influence AT performance. These factors that could alter antigen detection are also limitations that apply to molecular diagnostics, where ongoing monitoring of performance and vigilance for unexpected results are required. To date, both the original SARS-CoV-2 Delta strain and its descendant Omicron lineages have been detected with comparable efficiency across ATs. From the list of virus lineages we reported, we did not detect differences in AT performance. Kinetics of binding or other more sensitive analysis was outside the scope of this paper. Under the assumption of a relatively genetically uniform circulating viral strain, PCR Ct-based adjustments provided a pragmatic and scientifically valid strategy to reduce bias in estimating AT performance. In contexts where variant heterogeneity becomes relevant, the proposed distribution balancing approach could be extended by calculating variant-specific PPAfs and integrating them into proportionally weighted models. While Ct values serve as quantitative proxies with platform-dependent variability, internal laboratory calibrations (ie, processing of all nasal swabs within the same laboratory and RNA extraction and PCR protocol) mitigate this source of error. In the study, a single RNA extraction and PCR protocol of the Clinical Laboratory Improvement Amendment generated Ct values, thereby minimizing variability and controlling for recalibration effects.

In summary, we propose a robust statistical framework that corrects for real-world sampling biases through distributional modeling. This approach yields adjusted sensitivity estimates that more accurately reflect intrinsic test performance, thereby supporting improved diagnostic evaluation, regulatory decision-making, and public health comparisons across populations and settings.

## Abbreviations

AT: antigen test
Ct: cycle threshold
IRB: institutional review board
PDF: probability density function
PPA: percent positive agreement
PPAf: percent positive agreement function
qRT-PCR: quantitative reverse transcription polymerase chain reaction
